# Cancer research by means of tissue engineering – is there a rationale?

**DOI:** 10.1111/jcmm.12130

**Published:** 2013-10-01

**Authors:** Raymund E Horch, Anja M Boos, Yuan Quan, Oliver Bleiziffer, Rainer Detsch, Aldo R Boccaccini, Christoph Alexiou, Jiaming Sun, Justus P Beier, Andreas Arkudas

**Affiliations:** a Department of Plastic and Hand Surgery and Laboratory for Tissue Engineering and Regenerative Medicine, University Hospital Erlangen, Friedrich Alexander University (FAU) Erlangen-NurembergErlangen, Germany; b Emerging Fields Initiative, FAU Erlangen-NurembergErlangen, Germany; c Department of Plastic and Reconstructive Surgery, Tongji Medical College, Union Hospital, Huazhong Science and Technique UniversityHubei, China; d Department of Materials Science and Engineering, Institute of Biomaterials (WW7), FAU Erlangen-NurembergErlangen, Germany; e Department of Oto-Rhino-Laryngology, Head and Neck Surgery, Else Kröner-Fresenius-Stiftung-Professorship, Section for Experimental Oncology and Nanomedicine University Hospital ErlangenErlangen, Germany

**Keywords:** Tissue engineering, cancer research, tumourgenesis, tumour cells, cell transplantation, tumour growth, metastasis, AV loop, angiogenesis, telocytes, *in vitro* tumour models

## Abstract

Tissue engineering (TE) has evoked new hopes for the cure of organ failure and tissue loss by creating functional substitutes in the laboratory. Besides various innovations in the context of Regenerative Medicine (RM), TE also provided new technology platforms to study mechanisms of angiogenesis and tumour cell growth as well as potentially tumour cell spreading in cancer research. Recent advances in stem cell technology – including embryonic and adult stem cells and induced pluripotent stem cells – clearly show the need to better understand all relevant mechanisms to control cell growth when such techniques will be administered to patients. Such TE-Cancer research models allow us to investigate the interactions that occur when replicating physiological and pathological conditions during the initial phases of replication, morphogenesis, differentiation and growth under variable given conditions. Tissue microenvironment has been extensively studied in many areas of TE and it plays a crucial role in cell signalling and regulation of normal and malignant cell functions. This article is intended to give an overview on some of the most recent developments and possible applications of TE and RM methods with regard to the improvement of cancer research with TE platforms. The synthesis of TE with innovative methods of molecular biology and stem-cell technology may help investigate and potentially modulate principal phenomena of tumour growth and spreading, as well as tumour-related angiogenesis. In the future, these models have the potential to investigate the optimal materials, culture conditions and material structure to propagate tumour growth.

- Introduction
- Types of TE platforms for cancer research
- Breast
- Skin
- Melanoma
- Endothelial cells and endothelial progenitor cells
- Tumour cell interactions with bone
- Prostate
- Liver and brain
- Female reproductive system
- Drug delivery with TE techniques
- Newly detected cell types with potential for TE and cancer research
- Summary

## Introduction

The shape of the population pyramid as a sign of age distribution has been changing rapidly over the last 100 years with an increasingly heavy top, suggesting a significantly increased expectation of life. Hence, questions of maintaining sufficient quality of life in the elderly gain evermore relevance, because any extension of the human life span inevitably comes along with progressive functional loss of organs and tissue wear out [1–4]. Naturally, impairment of vital organ functions (such as heart liver or renal failure) poses severe medical problems, whereas wear out failures of large or small joints for instance is not vital but also impairs the quality of life for the individual patient. To overcome this problem, many groups in the field of TE have therefore focused on the development of functional tissue and organ substitutes. This has led to the creation of multiple 3-D matrices and scaffolds to be seeded with various types of cells in the laboratory [5]. As a by-product, these new technologies turned out to be also attractive for other areas of research, which, instead of rebuilding organs, primarily aim to detect mechanisms of angiogenesis, tumourigenesis [6–8], tumour spread [9–11] and potential ways of fighting cancer cell growth with anti-cancer drugs [12, 13], or developing direct or indirect drug delivery systems [14] for cancer therapy [15–17]. Tissue engineering provides pathologically relevant culture conditions, improved handling and *in vivo* applicability using defined matrices, growth factors and cell types in three-dimensional culture models. Similar to these applications, the characteristics of bacteria in terms of adherence [18], spreading and ingrowth as well as novel tools to prevent microbial adherence can be studied using TE models [18]. This holds also true for the observation of embryonal and adult stem-cell behaviour within scaffolds [19]. To generate surrogate tissue by transplanting 3-D scaffolds seeded with human embryonic stem cells (hESCs) between the liver lobules of severe combined immunodeficient (SCID) mice, such systems have been studied to investigate the teratoma-forming potential [20].

## Types of TE platforms for cancer research

Angiogenesis is important for tumour growth and spreading. Most cancer cells show abnormalities in differentiation and proliferation. These cells secrete various growth factors (e.g. VEGF) leading to blood vessel induction. Latest studies show that tumours may have the ability to generate their own capillary network [21]. TE can provide a 3-D environment mimicking organs or tissues with or without vascular networks, but certain limitations remain that can only been eliminated by implanting directly into host organisms. Components and properties of the microenvironment such as extracellular matrix, adhesion integrins, tissue architectures and tissue modulus regulate growth, differentiation and apoptosis of cells. These properties control cell fate through complex signals that are influenced either by interactions between neighbouring cells or by stimulated cell-surface receptors [10]. Reciprocal growth factor exchange between endothelial and malignant cells within the tumour microenvironment may directly stimulate neovascularization; however, according to Buchanan *et al.,* the role of host vasculature in regulating tumour cell activity is not completely understood [22]. With regard to different cancers and malignant tumours that have been subject to research in combination with TE techniques, some of the recent developments are highlighted. Various types of bioreactors have been found to play a crucial role for studying complex 3-D interactions of stem or tumour cells under conditions that resemble the clinical situation [23].

## Breast

Yang and Burg established an *in vitro* engineered microenvironment as a tissue test system that combined heterocellular tumour spheroids, polymeric microcarriers and adipocytes, an abundant stromal cell type in breast tissue. They intended to investigate the behaviour of breast cancer cells in response to different environmental stimuli in a more relevant 3-D microenvironment [10] Their results showed that the engineered microenvironment could influence breast cancer cell proliferation, differentiation and migration. This was attributed by the authors to be related to multi-cellular interactions and changes in microenvironmental stiffness. Although their findings had to be further investigated to confirm the results, their conclusion that stromal cells such as adipocytes could play a critical role in the breast cancer process [10] is of principal interest for cancer research [9]. Other studies have found that breast cancer metastasis suppressor 1 regulates hepatocellular carcinoma cell 1 apoptosis *via* suppressing osteopontin expression [24]. Tools like the transcriptional characterization of mammary stem-like cells and their differentiation with induced gene expression patterns can be made more easily accessible for research on mammary stem-like cells when novel mammary stem-cell markers are identified [25].

## Skin

Colley *et al*. addressed the limitation of current models for oral squamous cell carcinoma (OSCC) research, which are based on 2-D culture systems and therefore lack the complex architecture of native tissue [26, 27]. Using a three-dimensional *in vitro* model of the oral epithelium that replicates tumour progression from dysplasia to an invasive phenotype, they investigated OSCC cell lines either after seeding as a cell suspension (D20, Cal27) or as multicellular tumour spheroids (FaDu) with oral fibroblasts on an acellular dermis. These tissue-engineered models were then compared with patient biopsies. Over time, Cal27 cells were noted to traverse the basement membrane and to invade the connective tissue to reproduce features of early invasive OSCC [26]. In addition, their observations on FaDu spheroids revealed that these cells produced a histological picture that mimicked a carcinoma *in situ* with severe cellular atypia juxtaposed to normal epithelium. From these observations, it can be concluded that it seems possible to establish *in vitro* models with a native morphological appearance and histological characteristics of dysplasia and tumour cell invasion by utilizing native dermis. Such models could facilitate study of the molecular processes involved in malignant transformation in skin cancer, invasion and tumour cell growth as well as offering a platform for *in vitro* testing of new treatments, diagnostic tests and drug delivery systems for OSCC.

## Melanoma

Marrero *et al*. described an *in vitro* 3-D model in which they utilized a synthetic microgravity environment to study cell interactions [28]. To overcome some of the disadvantages of common 2-D monolayer cell culture models, which have been successfully used to understand various cellular reactions that occur *in vivo*, they described a free-floating 3-D tissue model to establish tumour spheroids. Their bioreactor system is known as the High Aspect Ratio Vessel (HARVs) and was used to provide a microgravity environment. The authors observed the HARVs bioreactor without the use of Matrigel™ to promote the aggregation of keratinocytes that formed a construct, which served as a scaffold for the growth of mouse melanoma in a 3-D model. They could show that melanoma cells interact with one another displaying observable cellular morphological changes [28]. The goal of engineering 3-D tissue models for use with tumour cells might be the collection of new information about cancer development. In addition, 3-D models with tumour cells might gain new insights into the development of novel potential treatment regimens that can be translated to *in vivo* models while at the same time reducing the need of using laboratory animals [28].

## Endothelial cells and endothelial progenitor cells

**Fig. 1:**
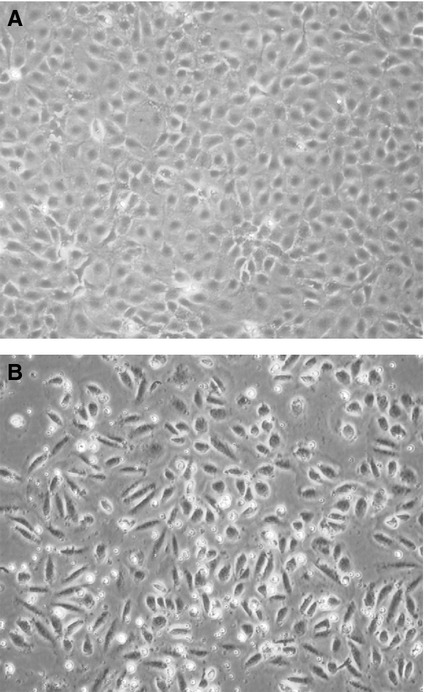
The rat endothelial cell line EC 52 was cultured either without (**A**: Control) or together with carcinoma cells, which were seeded in a cell culture insert on top of endothelial cells (**B**: indirect co-culture). Pictures were taken 1 week post-seeding.

**Fig. 2:**
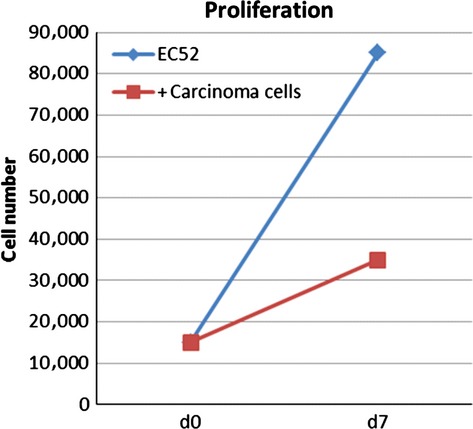
The rat endothelial cell line was cultured either without (Control) or together with carcinoma cells, which were seeded in a cell culture insert on top of endothelial cells (indirect co-culture). At the time-point of seeding, endothelial cells were plated into 6-wells at 60,000 cells per well followed by a cell count at 1 week after a week of culture or co-culturing, respectively.

When endothelial cell lines are inoculated with the supernatant of colon carcinoma and rhabdomyosarcome cell lines in a two-chamber cell culture insert systems or directly co-cultured with these cell lines, measurable interactions were seen with RNA profiling of the endothelial cells (Figs 1 and 2). Endothelial progenitor cells (EPC) have shown vascularization potential in ischaemic conditions and may also support blood vessel formation in tissue-engineered matrices. Bleiziffer *et al*. have investigated the impact of a well-characterized murine embryonal EPC line (T17b-EPC) on vascularization and fibrovascular granulation tissue formation using a 3-D fibrine matrix system that was subsequently subcutaneously implanted into an isolation chamber in rats. Endothelial progenitor cell were fluorescently labelled *in vitro* prior to implantation and fluorescence microscopy demonstrated integration of the transplanted cells in newly formed blood vessels within the fibrovascular granulation tissue adjacent to the fibrin clot [29]. It could be shown that EPC exhibited biological activity after subcutaneous implantation in a fibrin matrix and migrated from the fibrin clot into the granulation tissue and along intermuscular septae, undergoing differentiation into mature endothelial cells and integration into newly formed blood vessels and altering fibrovascular granulation tissue development. This corroborates the hypothesis that EPC may modulate the blood vessel formation in a bioartificial matrix [29, 30]. Gao *et al*. were able to show that tumours improve the expression of the transcription factor ld1 in EPCs. Furthermore, suppression of ld1 decreased tumour angiogenesis after metastatic colonization leading to impaired pulmonary macrometastasis and increase survival of animals [31]. To further improve the understanding of cell–cell interactions in tumour angiogenesis, the application of multiple cell types such as endothelial cells, tumour cells or stem cell in 3-D constructs is crucial.

## Tumour cell interactions with bone

**Fig. 3:**
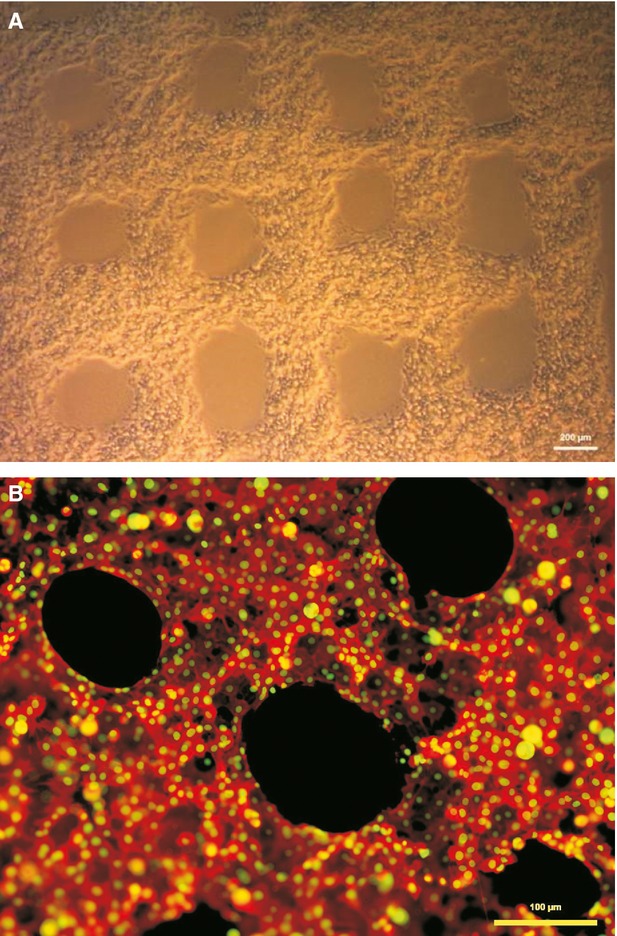
(**A**) Light microscopic image of hydrogel structures composed of alginate and gelatine with immobilized human osteosarcoma cells formed by bioplotting. (**B**) Fluorescence image of 3-dimensional plotted alginate-based hydrogel with osteosarcoma cells (nucleus in green and cytoskeleton in red stained) after 21 days of cultivation.

**Fig. 4:**
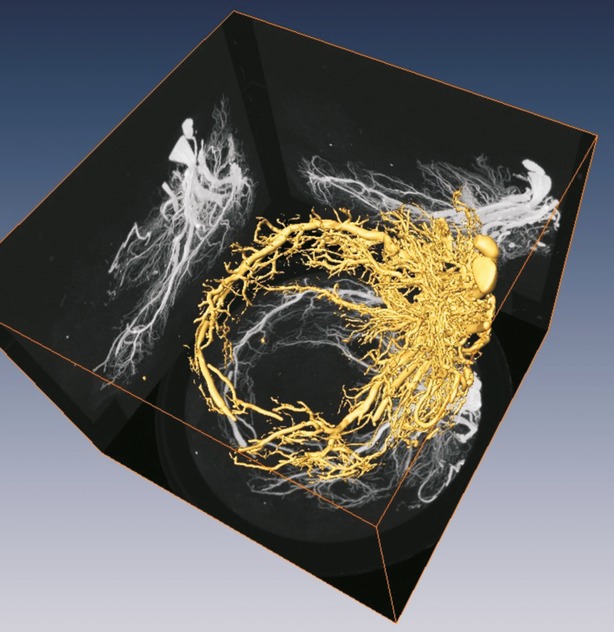
Micro-CT scanning of vascular sprouting from an arteriovenous loop in a tissue-engineered scaffold 4 weeks after implantation using fibrin gel immobilized VEGF165 and bFGF.

Bone TE is a very special field in tissue repair as unique physical properties differ from soft tissue research [32–36]. The combination of biodegradable polymers and bioactive ceramics in a variety of composite structures is a promising example in this area, while fabrication methods, associated cells and signalling factors determine the success of such strategies [36]. Another promising approach for the research of cell interaction with 3-D structures is cell printing [37]. Cell printing and bioplotting are rapid prototyping technologies for creating bioartificial tissue or organs of complex three-dimensional structure. One example from our emerging field initiative work is shown in Figure 3. The bioplotted hydrogel structures are composed of alginate and gelatin and containing embedded human osteosarcoma cells (Fig. 3a). The inner and outer geometry, pores and struts, should mimic the porous structure of bone tissue. After 21 days of cultivation, the hydrogel was completely penetrated by the embedded cells, which could create their own 3-D extracellular matrix (Fig. 3b). Therefore, the fabrication of cell-loaded constructs seems to be another helpful tool to study and to understand cell interaction in a bone-like structure in future. Mastro and Vogler described a specialized bioreactor based on the principle of simultaneous growth and dialysis that is designed to permit growth of 3-D, multiple-cell-layer osteogenic tissue from isolated osteoblasts over long-term continuous-culture intervals [38]. They analysed gene expression profiles and correlated them with cell-morphological changes over time, ultimately leading to increased expression of osteocyte-associated molecules. Their use of a bioreactor system provides an *in vitro* model that permits the study and manipulation of cancer cell interactions with bone tissue in real time [39, 40]. Metastatic human breast cancer cells, MDA-MB-231(GFP), introduced into the model colonized osteoblastic tissue in a manner that reflects various characteristics of pathological tissue observed in the clinical setting. While they found osteoblasts to be marshalled into a parallel alignment with cancer cells, followed by erosion of the structural integrity of the extracellular matrix, they noted that tissue degradation appeared to be accompanied by an increased expression of osteoblast inflammatory cytokines [38]. The addition of complex 3-D vascularization models using the av-loop technique within an isolation chamber *in vivo* has been demonstrated to successfully axially vascularize a clinically approved, mechanically stable bone substitute with a significant volume [34]. This could potentially be combined with drug loaded or modified osteoblasts in a tissue-engineered bone substitute for patients with bone defects after malignant tumour resection to transfer an axially vascularized and mechanically stable bone substitute with clinically relevant dimensions (Fig. 4) [34].

## Prostate

Long *et al*. described a capillary force-based method for seeding the human prostate cancer cell lines and stated that specific methods for efficient and reliable cell seeding into these and TE constructs used for regenerative medicine often remain poorly defined [41]. They seeded two different prostate cancer cell lines into sphere-templated poly(2-hydroxyethyl methacrylate) (pHEMA) hydrogels with static and centrifugation seeding. Whereas both cell lines attached and proliferated within the network, it was noted that histology revealed the formation of a necrotic zone by 7 days that was probably attributed to oxygen and nutrient diffusional limitations. Necrotic zone thickness was decreased by dynamically culturing cells in an orbital shaker. From their studies, they concluded that sphere-templated polymeric scaffolds could offer the potential to serve as an adaptable cell culture substrate for engineering a 3-D prostate cancer model [41]. Florczyk *et al*. addressed the treatment of castration-resistant prostate cancer (CRPC) that usually remains palliative [42]. They tried to establish a tumour model that would resemble the *in vivo* tumour microenvironment to investigate the interactions of CRPC cells with immune cells (human peripheral blood lymphocytes/PBLs) and other potential therapeutics. It was found that the use of 3-D natural polymer scaffolds can serve as a tissue culture model for supporting long-term analysis of interaction of prostate cancer tumour cells. That is in concordance with other observations where the clinically used marker prostate-specific antigen (PSA) was found to be extended 60 μm within static spheroids and was disrupted in mixed culture. Furthermore, spatial organization of the stained cells suggested a more aggressive form of cancer. The authors speculated that a higher cytokeratin expression could result from either differentiation towards a luminal phenotype or activation of the Ras pathway during dedifferentiation [43]. These findings are of interest because they contradict the existing paradigm of differentiation established for normal tissue. Cell dedifferentiation could be attributed to improved interstitial transport and synthesis of extracellular matrix in the spheroid TE tumour model [43]. These experiments show that TE techniques in tumour research can help elucidate the principal phenomena of prostate cancer cell development and could potentially provide *in vitro* platforms for rapid immunotherapy development [42].

## Liver and brain

Various scaffolds and models have been published with regard to liver cancer or brain cancer cell studies on the basis of different biomaterials for long-term culture of cells to study drug toxicity and hepatocyte metabolism in humans and develop a bioartificial liver (BAL) model [44]. Interest focuses on the potential utility of BAL as a bridge support for patients and as a module for experimental purposes. A radial-flow bioreactor (RFB), one of the perfused bed/scaffold-type bioreactors, enables a highly functional three-dimensional culture as BAL. The functional capacity of bioreactors depends not only on their mechanistic structures but also on scaffolds packed in them. In the present review, we examined the possible utility of a new porous organic-inorganic-hybrid scaffold in an RFB. The scaffold was made from tetraethoxysilane (TEOS) and polydimethylsiloxane (PDMS) by a sol-gel method using sieved sucrose particles as a porogen. In the porous TEOS-PDMS hybrid scaffold, human hepatocellular carcinoma cells (HepG2) proliferated actively and formed cell clusters more efficiently than they did in a polyvinyl–alcohol scaffold. When cultivated in PDMS-TEOS, HepG2 cells secreted an approximately threefold greater amount of albumin than those secreted in a monolayer culture. For potential application of BAL to pharmacological studies and future clinical use, it is essential to develop a method to propagate liver cells that maintain highly specific functions. Present results indicate that PDMS-TEOS may be a promising scaffold for developing such functional culture methods. Ma *et al*. developed a three-dimensional micro-scale perfusion-based two-chamber (3-D-muPTC) tissue model system to test the cytotoxicity of anti-cancer drugs in conjunction with liver metabolism [13]. When they cultured liver cells with different cytochrome P450 (CYP) subtypes and glioblastoma multiforme (GBM) brain cancer cells in two separate chambers which were connected in tandem on a 3-D TE scaffold (biodegradable polylactic acid/PLA), they were able to test the cytotoxicity of anti-cancer drugs, including temozolomide (TMZ) and ifosfamide (IFO). The strong metabolism-dependent cytotoxicity to GBM that was observed could be interpreted as a more physiologically *in vitro* environment than known from current 2-D monolayers for testing metabolism-dependent toxicity of anti-cancer drugs. These authors speculate that the two-chamber perfusion-based model could therefore be used as an important platform to better predict drug dosing and schedule, which would be the future aim of a personalized medicine approach [13]. Wu *et al*. investigated the role of breast cancer metastasis suppressor 1 (BRMS1) in hepatocellular carcinoma (HCC) using organotypic cultures and found that the expression level of BRMS1 was significantly downregulated in HCC tissues. On the other hand, expression of BRMS1 in SK-Hep1 cells did not affect cell growth under normal culture conditions, but sensitized cells to apoptosis induced by serum deprivation or anoikis [24]. Kataoka *et al*. described an organic–anorganic hybrid scaffold for the culture of HepG2 cells in a bioreactor mimicking a BAL. This model was proposed to be utilized as a bridge support for patients and as a module for experimental purposes [45]. They used a radial-flow bioreactor (RFB), which is one of the perfused bed/scaffold-type bioreactors, that enables a highly functional three-dimensional culture as BAL. In a porous hybrid scaffold, human hepatocellular carcinoma cells (HepG2) proliferated actively and formed cell clusters more efficiently than they did in a polyvinyl–alcohol scaffold. When cultivated in PDMS-TEOS, HepG2 cells secreted an approximately threefold greater amount of albumin than that secreted in a monolayer culture [45]. This model can serve not only for potential application of BAL to pharmacological studies but could easily be translated to a future clinical use [45]. Another approach has been published by using polymeric nanoparticles that are engineered to deliver siRNA and DNA to human brain cancer cells. Hydrolytically degradable polymers have been shown to effectively deliver DNA, whereas bioreducible degrading polymers effectively deliver siRNA [46]. On a cellular level, authors have also aimed to deliver a cargo to a particular intracellular site, if possible, to exert a local action. Nanovehicles (NV) are defined as a wide range of nanosized particles leading to colloidal objects, which are capable of entering cells and tissues and delivering a cargo intracelullarly. Prokop and Davidson have published the ‘elementary’ phenomena related to different levels of complexity of drug delivery to cancer and have underlined the importance of multi-scale modelling and bottom-up systems biology approach [47]. In brain cancer models, the simulation of the blood–brain barrier might be a challenge *in vitro,* but could be vital to realize the potential of large molecular weight substances to treat neurological disorders [48].

## Female reproductive system

Adissu *et al*. have published an *in vitro* 3-D cell culture model that should mimic the 3-D tissue organization and function [49]. Studies with epithelial cells from organs of the female reproductive system including the mammary glands, the uterus and the ovaries have shown that replicating normal tissue organization in the resting phase can be regarded as a prerequisite for appropriate physiological and pathological investigations, such as basoapical polarity of cells in 3-D cell cultures. They conclude that such models would help clarify detailed aspects of normal epithelial organization and function with regard to the extracellular matrix influences [49] and would allow for suitable applications of such systems in reproductive biology. Because of the ease of use and the consecutive clinical hype of retransplantation of aspirated autologous fat to augment or reconstruct breast tissue, adipose-derived stem cells (ADSC) have gained interest for research and for other potential clinical applications, such as wound healing studies. Adipose-derived stem cells have been postulated to be helpful in burn wound repair and regeneration [50]. To further investigate TE cell models, researchers have specifically cultured mammary epithelial cells derived from human breast hypertrophy patients in high purity to investigate possible pre-cancerous effects of these clinically relevant entity [51].

## Drug delivery with TE techniques

Polymeric materials have been used in a range of pharmaceutical and biotechnology products for several decades [52]. Primarily for reasons of clinical applicability, such materials were derived from various biodegradable products such as resorbable sutures, orthopaedic implants, macroscale and microscale drug delivery systems, such as microparticles and wafers used as controlled drug release depots [52]. One special focus that has emerged from TE research is the development of nanoscale drug delivery systems using liposomes and nanoparticles to facilitate the rational delivery of chemotherapeutic drugs in the treatment of malignant diseases. Recent developments have led to multifunctional nanoparticles (NPs) capable of targeting and controlled release of therapeutic and diagnostic agents. Among the published methods, short-peptide-based molecular hydrogels formed by biocompatible methods have been claimed to hold a potential for TE and controlled drug delivery [14]. Using a strategy of disulphide bond reduction and assistance with specific protein–peptide interactions, they expanded the applications of hydrogels into the fields of controlled stem-cell differentiation, cell culture and surface modifications of polyester materials by molecular self-assembly and anti-degradation of recombinant complex proteins to create a carrier-free system for long-term delivery of therapeutic agents [14]. Dhar and coworkers described the engineering of aptamer (Apt)-targeted nanoparticles (PLGA-b-PEG) encapsulating a pro-drug, which targeted the extracellular domain of the prostate-specific membrane antigen (PSMA), for enhanced *in vitro* cytotoxicity. They could demonstrate enhanced *in vivo* pharmacokinetics biodistribution, tolerability and efficacy when compared with cisplatin administered in its conventional form in normal Sprague Dawley rats, Swiss Albino mice and the PSMA-expressing LNCaP subcutaneous xenograft mouse model of PCa, respectively [53]. By yielding significant dose-sparing characteristics of their test drug, with equivalent anti-tumour efficacy in LNCaP xenografts at one-third of the dose of cisplatin administered in its conventional form, their findings could provide a remarkable improvement in the drug therapeutic index with TE techniques [53]. Kamaly *et al*. postulated that new generations of targeted and controlled release polymeric NPs, which can be engineered to navigate the complex *in vivo* environment, may offer additional therapeutic properties. Furthermore, they might incorporate functionalities that can achieve target specificity, control of drug concentration and exposure kinetics at the tissue, cell and subcellular levels [52]. Tissue engineering platforms could optimize conventional methods of pharmacology when multifunctional NPs are designed. This could lead to improved drug safety and efficacy, and may be complementary to drug enhancements that are traditionally achieved by medicinal chemistry, creating a new class of therapeutics, distinct from the original active drugs used in their composition, and distinct from first-generation NPs that largely facilitated drug formulation [52]. Liposome technology has been described to offer improved treatment results of multidrug-resistant cancers while at the same time reducing cardiotoxicity [54]. Kelleher and Vacanti have suggested that nanotechnology may be of use in breeching the barriers to commercialization of TE technologies, although its primary mission is to improve the technology by solving some remaining and vexing problems in its science and engineering aspects [55]. Nanoparticles have been combined with superparamagnetic iron oxide nanoparticles (SPION) to be used for diagnostics and therapy (‘theranostics’). Alexiou *et al*. have found that SPION, which is already used as contrast agents in magnetic resonance imaging, can be utilized *in vitro* to separate cells and for magnetic drug targeting. For advanced multimodal therapies such as hyperthermia plus irradiation, SPION can be heated by alternating magnetic fields [56]. However, to exclude possible toxic side effects, further studies seem to be warranted before the clinical translation into human organisms can be safely performed [57–59].

## Newly detected cell types with potential for TE and cancer research

**Fig. 5:**
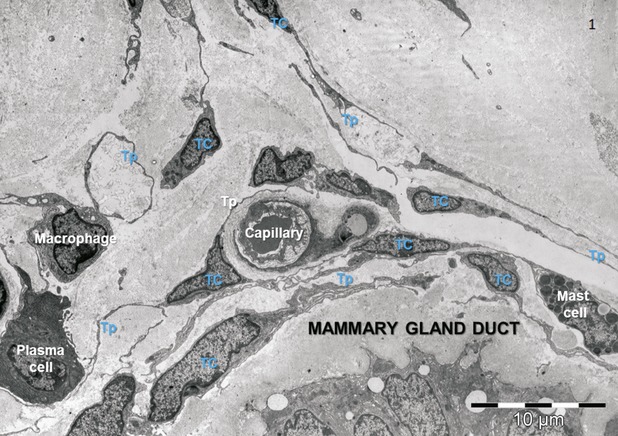
Transmission electron microscopy, human mammary gland (peri-tumoural zone). At least six telocytes (TC) with their corresponding telopodes (Tps), surrounding a blood capillary, cross-sectioned. Courtesy to Drs. Mihaela Gherghiceanu and L.M. Popescu, National Institute of Pathology, Bucharest, Romania.

**Fig. 6:**
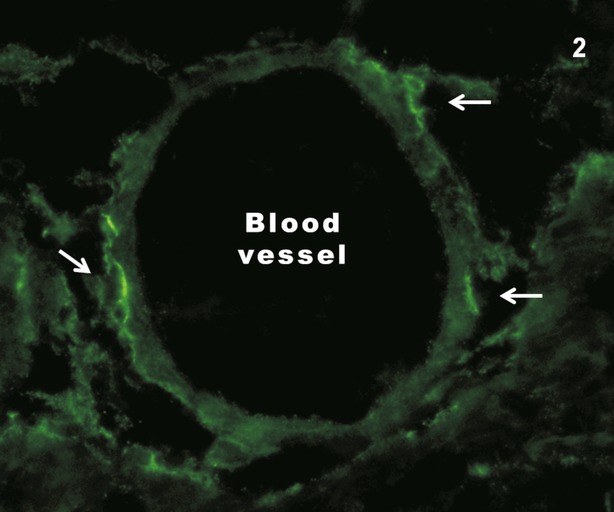
Immunofluorescence for PDGFR beta (platelet-derived growth factor receptor beta) typical positive expression in TC (arrows), in the outer layer of the blood vessel (Objective: 40×). Courtesy to Drs. Laura Ceafalan and L.M. Popescu, National Institute of Pathology, Bucharest, Romania.

As a result of the increasing capability to culture various cell types and to manipulate cells under culture conditions in different ways, knowledge has been gained about their specific cellular and molecular functions (Figs 5 and 6). In contrast to *in vivo* situations, laboratory conditions can be modified in many ways. This allows researchers to set up specific conditions to study cell metamorphosis, apoptosis and differentiation and dedifferentiation. Similar to previous dogmas that formerly seemed to be untouchable – such as the DNA-RNA-protein sequence – and that had to be revised, it seems also notable that previously, tissue that was deemed unimportant in terms of carcinogenesis rather than the pure organ malignancies is gaining more attention now. This is especially true for the interstitial tissue that has long been neglected in cancer research.

The biochemical microenvironment of cells consists of cytokines, growth factors and other biomolecules, leading to complex signalling pathways. Most cells attach to the extracellular matrix *via* cell-surface integrins, transducing signals from the matrix site to the cell. Studies are conducted to observe effects of combining soluble factor signalling and cell–matrix interactions on cell behaviour. These studies are important to optimize the cell microenvironment in TE applications and for further cancer cell research. Various groups have studied cells in the interstice and there is growing evidence that these cells may play a crucial role in tissue regeneration as well as potentially in cancerogenesis [60–64]. Cells that had formerly been described as interstitial Cajal like cells have gained international attention when within the last years the term ‘telocytes’ was coined [65]. Telocytes have been reported in various tissues [62, 64, 66–71]. Ultrastructural techniques reveal a complexity in interstitial cell populations with remarkable changes in disease.

Telocytes were found with significantly higher numbers in the epicardium than in the myocardium and they were distributed longitudinally and within the cross network of myocardium [72]. As isolation of telocytes and propagation in culture have been possible, *in vitro* experiments have shed more light on possible functions of these interstitial cells. Cardiac infarction has been of special interest with regard to regeneration because of the vital consequences of functional loss when myocardial tissue is lost. Molecular analysis of cardiac stem cells for cardiac regeneration is believed to be instrumental to establish cardiac regeneration technologies [73, 74]. Researchers have investigated the close relationship between telocytes and cardiac stem cells. In terms of RM, first experiments from different groups are on the way to identify a proposed functional impact of telocytes when injected into tissue damage zones. Simultaneous transplantation of cardiac telocytes and mesenchymal stem cells decreases peripheral fibrosis of the border zone when directly injected. Rebuilding the cardiac telocyte network deceased infarction size and improved myocardial function [72].

Telocytes form a cardiac interstitial network involved in the long-distance intercellular signalling coordination [68]. From intense ultrastructural studies, it has been claimed that the telocytes' cardiac network shapes the structural support as a prerequisite to integrate the overall information from the vascular system (endothelial cells and pericytes), nervous system (Schwann cells), immune system (macrophages, mast cells), interstitium (fibroblasts, extracellular matrix), stem cells/progenitor cells and working cardiomyocytes. This integrative interstitial system might sustain a coordination of multicellular signals, essential for cardiac renewal, regeneration and repair [68].

Although the isolation and culturing of telocytes are still tedious, the principal possibility is now given to combine telocyte cultures with common TE techniques and appropriate scaffolds to modulate and direct their growth. Given the unique properties of telocytes and their close relationship with organ-specific cells, it seems promising that the further studies of interstitial cells in combination with stem and tumour cells could open completely new ways and insights into carcinogenesis in the context of TE and RM experiments.

## Summary

In summary, this paper systematically seeks to comment some of the recent developments in utilizing TE platforms for cancer research. The evolution from 2-D to 3-D cell cultures and tandem bioreactors to investigate tumour cell growth, tumour-related angiogenesis, attachment and spreading of tumour cells in TE matrices, as well as the prospect of modulating these phenomena in a nature mimicking environment in combination with supramicrosurgical methods of vascularization, opens new horizons for anti-cancer research.
